# Hospital Economics.—V. The Statistical Report of King Edward's Hospital Fund


**Published:** 1914-03-07

**Authors:** 


### HOSPITAL ECONOMICS.

### V. The Statistical Report of King Edward's Hospital Fund.*

Ten years ago it was said by one who was well qualified to judge that for the hospitals of London to be brought into line in matters of reform the influence of a body possessing the power of the purse must make itself felt. Since that utterance the history of the institutions referred to has proved the truth of the dictum.

The power of the purse was brought to bear. The Central Funds required the presentation of statistics and accounts in a certain form before grants could be considered. This decision was irksome at first; and even now, whilst loyally working on the plan laid down for their guidance, the whole of the London hospitals are not in agreement as to the complete justice of some of the regulations and of the allocation of certain items in the index of classification. But on the whole it is safe to say that there is practically a unanimous consensus of opinion proclaiming the benefits of a system by which the hospitals are able, through the provision of accurate records for the purpose of comparison, to learn from each other.

### A King's Fund for the Provinces.

Where London was ten years ago the provinces are to-day. Would it be possible to exercise on their behalf the same kind of influence for the same end? To do it would be a giant's task, involving a federation of Hospital Sunday and Hospital Saturday Funds acting in conjunction with the League of Mercy, and even then the operations of such a combination would leave some parts of the country untouched. But is it necessary? Cannot the lesson of London be brought home to the provincial hospitals in each large town on the ground of economy?

When the writer of these articles was, a few years ago, asked to report on the management and financial position of one of our provincial hospitals, he found great difficulty in comparing the accounts and statistics with the corresponding figures of hospitals of similar size and character. The general arrangement of the income and expenditure accounts looked at was certainly in many cases roughly on the lines of the Uniform System in its earlier form, but the variations from the model were so important that to compare the figures with any confidence as to the reliability of results was impossible.

### Provincial Hospital Accounts.

After a lapse of a few years, examination of the reports of a rather larger number of English and Scottish hospitals reveals the fact that no steps have been taken on any large scale in recent years to bring about the use of a uniform system by provincial hospitals. It appears, moreover, that divergence of method is not only in respect of the form of accounts, but of the period to which they refer; the majority have a financial year concurrent with London, but in a number of places, for the purposes of the statistics, the year commences at such varied times as March 1, May 16, June 1, and August 1.

It is not possible here to say anything in respect of the preparation of the records of the numbers and attendances of patients in these hospitals, as the methods by which the results have been obtained are not apparent. As any statements of averages are entirely governed by these figures, unless one is able to be sure that they have all been calculated in an identical manner, it is idle to speculate on the results shown. The financial report itself is, of course, capable of being more closely examined, and in many cases the hospitals reviewed prepare their accounts roughly in the form formerly employed by most hospitals, but amongst this group a large variation of arrangement is still to be found. An instance can be given demonstrating how departure from an index of classification renders comparison futile. It is the case of an important Yorkshire hospital, where large expenditure clearly belonging to the maintenance of the building is put down under extraordinary expenditure, thereby considerably reducing the cost per occupied bed.

### Why not the Uniform System?

In quite a number of other places the statements are prepared in what often amounts to an incoherent form. Practically all the hospitals publish a list of investments, but very few a balance sheet.

Something, however, has been gained by the provincial hospitals through this delay in common action, for they are now in a position to profit by the sound lessons taught by the experience of the Metropolis. It is easy to recognise the difficulties of the provinces in coming to an agreement as to a uniform statistical plan. The institutions are widely separated and conference is not feasible. Therefore, it seems reasonable to suggest that the form of accounts, as laid down by the London Central Funds, might be, at least tentatively, adopted.

If such a desirable state of things can be brought about, the excellent results of the Statistical Report of King Edward's Hospital Fund for London will prove even more far-reaching and valuable.

### ASSISTANT MENTAL MEDICAL OFFICERS.

Dr. F. J. Stuart, Hon. Secretary of the Association of Assistant Medical Officers of Mental Hospitals, England and Wales, asks us to state that the first annual general meeting of the Association will be held at the Westminster Palace Hotel, London, on Saturday, March 14, at 3.15 p.m.

* Previous articles appeared on November 29 and December 13, 1913, and January 17 and 31, 1914.

